# Influence of curing protocol and ceramic composition on the degree of conversion of resin cement

**DOI:** 10.1590/1678-7757-2016-0270

**Published:** 2017

**Authors:** Marcos Daniel Septimio Lanza, Marcello Rubens Barsi Andreeta, Thiago Amadei Pegoraro, Luiz Fernando Pegoraro, Ricardo Marins De Carvalho

**Affiliations:** 1 Universidade Federal de Minas Gerais, Departamento de Odontologia Restauradora, Belo Horizonte, MG, Brasil.; 2 Universidade Federal de São Carlos, Departamento de Engenharia de Materiais, Laboratório de Materiais Vítreos, São Carlos, SP, Brasil.; 3 Universidade do Sagrado Coração, Departamento de Prótese e Implantodontia, Bauru, SP, Brasil.; 4 Universidade de São Paulo, Faculdade de Odontologia de Bauru, Departamento de Prótese e Periodontia, Bauru, SP, Brasil.; 5 University of British Columbia, Faculty of Dentistry, Department of Oral Biological and Medical Sciences, Division of Biomaterials, Vancouver, Canada.

**Keywords:** Resin cement, Micro-Raman spectroscopy, Degree of conversion, Ceramics

## Abstract

Due to increasing of aesthetic demand, ceramic crowns are widely used in different situations. However, to obtain long-term prognosis of restorations, a good conversion of resin cement is necessary.

**Objective:**

To evaluate the degree of conversion (DC) of one light-cure and two dual-cure resin cements under a simulated clinical cementation of ceramic crowns.

**Material and Methods:**

Prepared teeth were randomly split according to the ceramic's material, resin cement and curing protocol. The crowns were cemented as per manufacturer's directions and photoactivated either from occlusal suface only for 60 s; or from the buccal, occlusal and lingual surfaces, with an exposure time of 20 s on each aspect. After cementation, the specimens were stored in deionized water at 37°C for 7 days. Specimens were transversally sectioned from occlusal to cervical surfaces and the DC was determined along the cement line with three measurements taken and averaged from the buccal, lingual and approximal aspects using micro-Raman spectroscopy (Alpha 300R/WITec^®^). Data were analyzed by 3-way ANOVA and Tukey test at =5%.

**Results:**

Statistical analysis showed significant differences among cements, curing protocols and ceramic type (p<0.001). The curing protocol 3x20 resulted in higher DC for all tested conditions; lower DC was observed for Zr ceramic crowns; Duolink resin cement culminated in higher DC regardless ceramic composition and curing protocol.

**Conclusion:**

The DC of resin cement layers was dependent on the curing protocol and type of ceramic.

## Introduction

All-ceramic restorations have become popular for excellent aesthetics, color stability, abrasion resistance and biological compatibility^2^. However, to obtain restorations with favorable prognosis, a high degree of conversion (DC) of resin cement should be achieved^1^ ^,^ ^21^. Incomplete polymerization of the resin cement occurs by decreased energy from the light source through the ceramic material. The amount of this attenuation has been shown to be directly dependent on the composition, thickness, opacity and color of the materials used as restoration^2^.

There are different types of ceramics used in dentistry, with different degrees of translucency influencing the depth of cure and hardness of resin cements as a function of light attenuation^21^. The decrease in light intensity can be caused by absorption and scattering of light by physical and structural differences of the restorations^1^. Ceramic restorations are considered optical heterogeneous materials^23^ with varying degrees of translucency that can be changed by the thickness, crystal structure, porosity between the layers, possible change in the constitution of infrastructure, and a reflection of the infrastructure between the interface and the ceramic cover^13^ ^,^ ^14^. Furthermore, increased thickness more than 2 mm has a significant effect on the transmission of light and, consequently, the hardness of resin cements^1^ ^,^ ^17^. Other factors that can influence the polymerization of resin cements are related to the energy intensity of the light source, wavelength, time and distance from the energy source in relation to the composite^5^ ^,^ ^18^ ^,^ ^30^. Peutzfeldt and Asmussen^25^ (2005) stated that the amount of energy has significant influence on the DC, the extent of crosslinking and the physical and mechanical properties of the composite being cured.

Dual-cure resin cements have been developed under the concept that chemical cure alone could achieve substantial polymerization in areas unreachable by the light source. The light-cured portion should optimize the cure along the exposed cement layer and provide initial stability until the self-curing could mature. However, it is known that the two curing routes are independent and the rate as to each one cures varies among materials and is rather an inherent aspect of their chemistry. Considering this, we may say that the curing rate of some cements can be more dependent on light exposure than others^20^ ^,^ ^22^ ^,^ ^24^.

Among the methods used to determine the DC of composites, the Raman spectroscopy has been shown to be very suitable for being relatively simple, reproducible, noninvasive, and for allowing the use of thin specimens without requiring special preparation^26^. Some studies have evaluated the DC of resin cements when cured through ceramic slabs^17^. However, there is lack of data on the DC of resin cements when cured through actual crowns, under a simulated clinical setting. This is relevant because under a crown, the cement layer is covered by a varying thickness of ceramic from cervical to occlusal surfaces. Moreover, limited access and the presence of neighboring teeth in the mouth lead clinicians to decide for a curing protocol of the exposed ceramic surfaces, without actually knowing if the DC would be affected. Thus, this study aimed to evaluate the influence of activation protocols and different composition of ceramics in the DC of resin cements under a simulated clinical setting. The following null hypotheses were tested: (1) the curing protocol does not affect the DC of the cements; (2) the ceramic composition has no influence on the DC of the cements.

## Material and methods

### Preparation of the samples

This study was approved by the Bauru School of Dentistry, University of São Paulo (protocol no. 150/2010). Thirty-six sound human premolars that were extracted for orthodontic reasons received fullcrown preparations adequate for ceramic restorations (1.5 mm axial and 2 mm occlusal reduction; and 6° and 10° of double convergence). Impressions were taken from the preparations (Impregum/3M ESPE, St Paul, Minnesota, USA) and individual dies created with gypsum (Type IV Velmix Kerr, Romulus, Michigan, USA). The dies were randomly divided into two groups (n=18). One group was used to construct monolithic ceramic crowns in lithium disilicate (LD) glass ceramic (IPSe.maxPress/IvoclarVivadent, Schaan, Liechtenstein, Germany). The other group was used to construct infrastructures of high-strength zirconium oxide (IPSe.maxZirCAD/IvoclarVivadent, Schaan, Liechtenstein, Germany), with 0.5 mm thick and 70 pm space for cement, followed by veneering with pressed ceramic-based fluorapatite oating (IPSe. maxZirPress/IvoclarVivadent, Schaan, Liechtenstein, Germany). For both ceramic types, the thicknesses of the final crowns were standardized at 1.5 mm and 2.0 mm for axial and occlusal surfaces, respectively; and were constructed in shade A2.

The fitting of the crowns was tested in each preparation with the aid of low viscosity silicone (Xantopren^®^ VL Plus/Heraus Kulzer, Hanau, Hesse, Germany). Contact areas were removed by reducing the preparation to avoid altering the thickness of the crown. The fitting test was repeated until a uniform film of silicone was obtained. Cervical adaptation was assessed with the aid of an explorer #05 (Hu-Friedy, Chicago, Illinois, USA) by an independent operator and was considered acceptable when the probe did not detect discrepancies at the crown-tooth interface.

### Cementation and curing protocol

**Figure 1:**
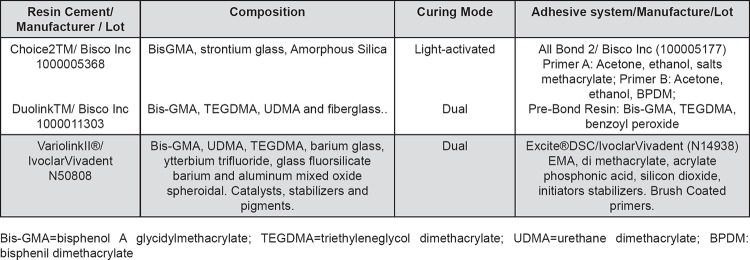
Cements and adhesive systems used in this study

The materials used in this study are listed in Figure 1. The criteria used to choose these materials were based on manufacturer's indication for all ceramic crowns cementation (two dual cured resin cement), and to validate our methodology as a negative control (one light activated resin cement).

**Figure 2:**
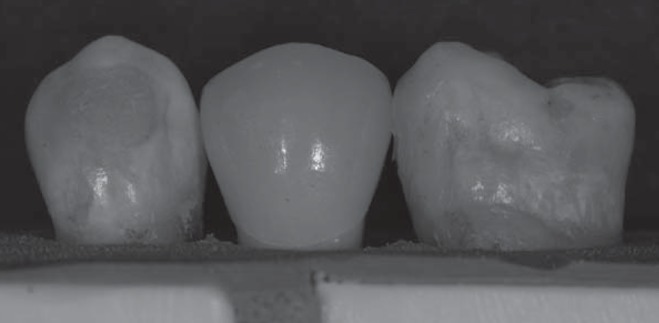
Clinical setting with two natural extracted teeth and the prepared teeth after the crown has been cemented

**Figure 3:**
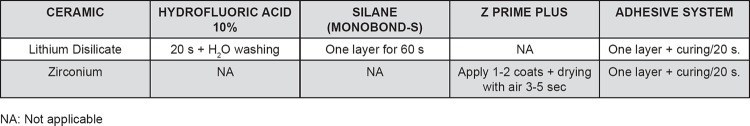
Bonding procedures for the ceramic crowns

**Figure 4:**
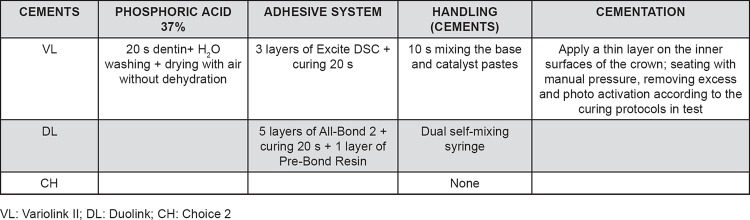
Bonding procedures for dentin and cementation

To simulate the clinical setting, two natural, extracted teeth (one premolar and one molar) were fixed by the roots on a putty PVS (Optosil^®^/Heraus Kulzer, Hanau, Hesse, Germany) mold allowing a distance between each other to fit the prepared teeth to receive the cementation. The distance was set in a manner that there would be proper interproximal contact of the natural teeth and the crown being cemented (Figure 2). Before cementation, the preparations were cleaned with pumice slurry (SS White Dental Articles Ltda, Rio de Janeiro, RJ, Brazil), washed in running water and dried with air for 30 s. For Choice 2 and Duolink resin cement, the adhesive system All Bond 2 (Bisco/Inc^®^, Chicago, Illinois, USA) was used, followed by PrimeBond (Bisco/Inc^®^, Chicago, Illinois, USA) application. For VariolinkII resin cement, the adhesive system ExciteDSC (IvoclarVivadent, Schaan, Liechtenstein, Germany) was used. The prepared teeth were fixed in the space between the natural teeth and both the dentin and the intaglio surface of the crown were treated according to the respective manufacturer's directions (Figures 3 and 4).

Light curing of resin cement was performed (LED; Ultraled Dabi Atlante, Ribeirão Preto, SP, Brazil) with output power density of 950 mW/cm^2^, monitored for consistency with the aid of a radiometer (Model 100, Kerr Corporation, Orange, California, USA) before each use. On some measures we detected a lower intensity compared with that provided by the manufacturer, but not clinically significant. The sample set was randomly divided into subgroups (n=3), according to the cement and activation protocol selected, as follows:

1. Curing Protocol 3x20: photoactivation in the buccal, lingual and occlusal surfaces, with exposure time of 20 s on each side, sequentially;
2. Curing Protocol 1x60: photoactivation only by occlusal surface with exposure time of 60 s.

After cementing, the set was left undisturbed for 15 mi and then stored in deionized water at a constant temperature of 37°C for 7 days in a dark environment. After this period the specimens were serially sectioned (Isomet 2000 precision saw - Buehler, Lake Bluff, Illinois, USA) in three regions: 2 mm below the occlusal surface plane; at the center of the crown; and 2 mm below the cervical margin, to obtain two discs of approximately 2 mm thickness. The discs were manually polished for 1 minute using 1200 and 2000 grit SiC paper (EXTEC - Erios Internacional, São Paulo, São Paulo, Brazil), and ultrasonicated for 5 min between each, and after polishing. The experimental design was performed in order to evaluate the behavior of resin cements and to read the whole area filled by resin cement.

### Degree of conversion analysis

**Figure 5:**
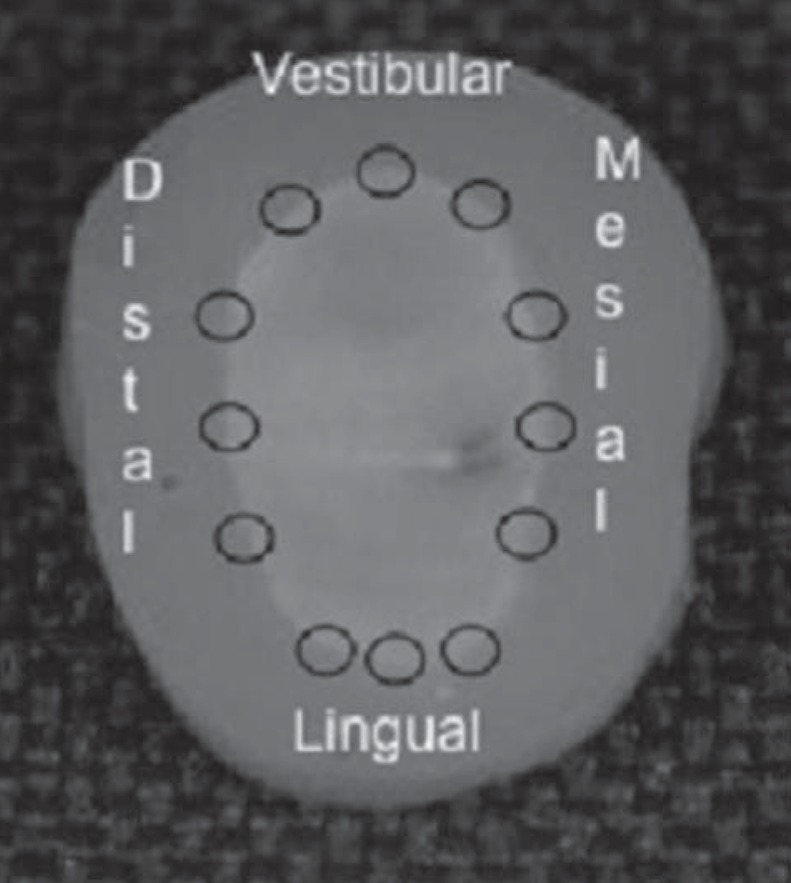
Schematic diagram representing reading location related to the proximal aspects

**Figure 6:**
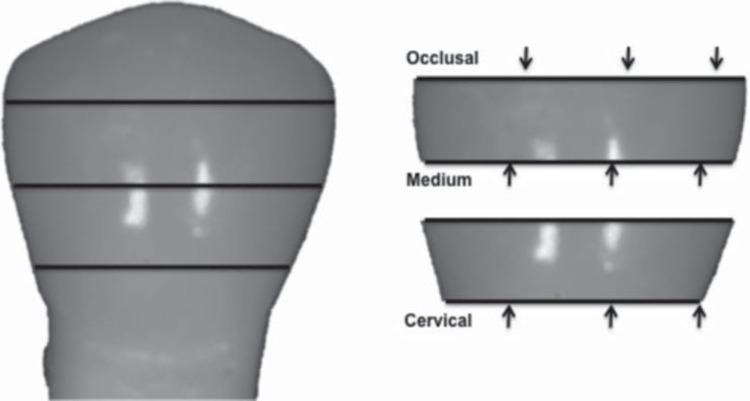
Diagram illustrating the transversal cuts and discs, pointing the sides that represented the occlusal, medium and cervical thirds

The DC was analyzed in a micro-Raman combined with a confocal optical microscope (Alpha 300 A/R-WITec^®^, D-89081 Ulm, Germany), composed of a laser He:Ne with a wavelength of 632 nm. Each disc was placed on the platform of the microscope to locate the line of the cement. Twelve readings were performed on each disc, three in each side (buccal, mesial, lingual and distal) of each third (occlusal, medium and cervical) as total of 108 readings for each experimental group (Figures 5 and 6, respectively). To allow for the calculation of the degree of conversion, samples of the uncured cements were manipulated, immediately dispensed in a prefabricated stainless steel matrix (4 mm x 1 mm) and the readings performed with the Raman instrument.

The Raman instrument was aligned for measuring the relative intensity of the aromatic band, with the main peak at 1608 cm^-1^, relative to the band with aliphatic major peak at 1638 cm^-1^. All Raman measurements were taken with four acquisition spectra of 20 s each. The determination of DC was performed in accordance with the maximum peak at 1638 cm^-1^ compared with the peak 1608 cm^-1^, according to the equation:

$$\text{DC}(\%)=100\times [1-{\text{R}}_{\text{cured}}/{\text{R}}_{\text{uncured}})]$$

where R represents the ratio between 1638 cm^1^/1608 cm^-1^ of polymerized and unpolymerized cement^15^.

### Statistical analysis

Data were analyzed by 3-way ANOVA (Sigma Plot 12.0, Jandel Scientific, San Rafael, California, USA), for factors cements X curing protocols X ceramics, and Tukey's test for all pairwise multiple comparison with a significance level of α=5%.

## Results

The average results (%) DC and standard deviations of the groups evaluated are shown in Table 1. Statistical analysis showed significant differences for cements (F=395.92, p<0.001), ceramics (F=363.95, p<0.001), and curing protocols (F=148.30, p<0.001), with a statistically significant interaction between factors (F=9.679, p<0.001). Pair-wise comparisons showed that the DC of the two dual-cure resin cements (Duolink/Bisco/Inc^®^, Chicago, Illinois, USA and Variolink II/IvoclarVivadent, Schaan, Liechtenstein, Germany) was always significantly higher under lithium disilicate crowns, regardless of the curing protocol used. The exception was observed for the light-cured resin cement (Choice 2) for which the differences were not significant. In general, the 3x20 curing protocol resulted in higher DC for each individual combination of ceramic and resin cement. This was even more evident for the light-cured only resin cement CH, and for the ZR ceramic system.

**Table 1:** Mean pooled values in %DC (SD)

	Curing Protocol					
	1X60n			3X20u		
Resin Cement	DL	VL	CH	DL	VL	CH
Ceramic						
DSLT	85.8(4.3)^A,a^	75.5(5.8)^A,b^	67.8(8.5)^A,c^	86.7(3.2)^A,a^	77.4(2.9)^A,x^	74.4(6.0)^A,y^
ZR	76.3(3.2)^B,a^	61.4(10.9)^B,c^	65.5(12.2)^A,b^	78.3(3.6)^B,ax^	70.8(3.9)^B,x^	72.2(6.7)^A,x^

Means followed by different symbol for curing protocol, upper case for ceramic (column) and lower case for resin cement (row) differ statistically by Tukey test (p<0.05).

CEMENTS: VL: Variolink II; DL:Duolink: CH:Choice 2.

CERAMICS: DSLT: Lithium Disilicate; ZR: Zirconium.

## Discussion

This study innovatively evaluated the DC of resin cements when cured at a clinical simulated setting. For this purpose, actual crowns were created from either lithium disilicate (DLST) or Y-TZP (ZR) and cemented on the originating preparation cut on extracted teeth, and respecting a clinical scenario where neighboring teeth are present. Thus, we evaluated two different curing protocols that would likely be chosen by clinicians in order to ensure the curing of the cement under the crowns. Without varying the total energy delivered, the cement was cured either by light exposing each one of the three surfaces available (buccal, lingual and occlusal) for 20 s (3x20) or delivering a single exposure of 60 s from the occlusal surface only. While the total energy delivered with both protocols remained unchanged, clinicians would tend to think that the multiple exposure protocol is more time consuming^8^, thus preferring the single exposure without knowing if this would affect the actual curing of different cements under different ceramic systems.

The results showed that the curing protocol influenced the DC. Therefore, the first hypothesis should be rejected. Similar findings were reported^10^ on microhardness of resin composites used in class II restorations and showed that the fractional photoactivation promoted superior results than those obtained with single photoactivation. When applying a single, large amount of power as the protocol 1x60, the monomers appear to be activated faster and generate multiple growth centers, which may cause premature polymerization of the composite due to the decrease in mobility of radicals, which, in turn, prevents migration of active free radicals and thus causes a low DC^11^ ^,^ ^12^. Conversely, if the reaction process is slower, the resinous material may be capable of remaining in state "fluid" for a longer time and provide higher DC due to the greater mobility that occurs between free radicals, which improves the efficiency of reaction^6^ ^,^ ^11^.

Another factor that may have contributed to the lower DC when the cements were activated by the occlusal surface only (1x60) was the distance from the tip to the most cervical regions of the crowns. Our experimental design allowed for determining the DC at different surfaces and thirds of the crowns (see Material and Methods). We observed that while there were no differences among the buccal, lingual and approximal aspects at each individual third, we did observe lower DC at the cervical third when the 1x60 curing protocol was used (data not shown) and that was more evident for the more opaque ZR system. Although the readings allowed for analysis of surfaces and thirds, and did provide the important information that cervical thirds are at higher risk of compromised curing when light activation is only delivered from occlusal surface, they are all regarded as correlated for statistical purposes and, therefore, were pooled for the Anal analysis. Resin cements have tapering polymerization according to the distance from light. Regarding curing protocol, Variolink and Duolink exhibited similar DC values when cured under lithium disilicate glass ceramic, suggesting that the distance from the light for those cements was not a relevant factor when using more translucent ceramic. This argument finds support in the literature^19^. Furthermore, Duolink also presented more uniform DC when cured under the more opaque ZR. It has been reported that Duolink has lower concentration of filler particles, and this may facilitate the mobility and reactivity of curing monomers, thus resulting in more uniform curing, regardless of the curing protocol and ceramic type^24^.

The optimum time of activation of different composites is not yet fully established. It is speculated that longer light exposures promote greater DC^9^. According to some authors^27^ ^,^ ^28^, a minimum of 60 seconds of photoactivation through ceramic restorations with thickness 2 mm or more would be needed to promote adequate curing of the resin cement. Furthermore, a decrease in the DC was observed with opaque ceramic when activated for 40 s^3^. In this study, we chose the total exposure time of 60 s, regardless of the curing protocol as an arbitrary time that would cover most of the concerns of studies and recommendations of manufacturers regarding the ideal curing time for resin composites. In addition, we assumed that 60 s would be a clinically justifiable exposure time when curing through ceramics.

Regarding the composition of the ceramics, our results showed a significant influence on the DC for most resin cements, thus leading us to partially reject the second hypothesis. In general, the DC was always higher for the lithium disilicate than for the zirconia-based ceramics. Glass-ceramics have fewer crystal structure and greater translucency when compared with alumina-based and zirconia-based ceramics^13^ ^,^ ^14^. Accordingly, the higher the light transmittance, the greater the polymerization of cement^21^. An exception occurred with the negative control, light-cured cement (Choice 2) that resulted in similar DC for both ceramics with no statistically significant difference. We expected that curing of this cement would be compromised by the imposed limitations of light transmittance. However, since this was not the case, we speculate that the uniform curing observed in Choice 2 light-cured cement was due to the application of Pre-Bond Resin after conditioning with All-Bond 2 adhesive (see Material and Methods). This step could enhance the DC along the interface because of the presence of benzoyl peroxide in the formulation of Pre-Bond^4^ ^,^ ^15^ ^,^ ^29^. This could also explain the more uniform DC observed for Duolink, which also includes Pre-Bond in the bonding procedure.

Because of inherent differences in the formulation and curing mode of the cements, it was not expected that they would have similar DC. However, *post hoc* test did not show difference between the light-cured (Choice 2) and the dual-cure (Variolink 2) resin cements when used to cement the Zr crowns and cured with the 3x20 s protocol. This can be explained by the rapid crosslinking polymer chain formation of some cements when exposed to light due to the high concentration of photo initiators, which considerably increases the radiation sensitivity, even when this is attenuated by the restorative material^15^. Furthermore, some studies have demonstrated that VL resin cement is very dependent of radiation to enhance optimal DC^15^, and, probably the lower DC could be related to the insufficient amount of light that reached the resin cement. In addition, as previously discussed, the application of Pre-Bond Resin for Choice 2 enhances the formation of free radicals that promotes increased DC^16^.

## Conclusions

(1) The curing protocol affected the DC of the resin cements. Superior and more uniform curing was achieved by the 3x20 protocol.

(2) The ceramic type/composition influenced the DC of the cements. Higher DC was obtained when the cements were used to cement the lithium dissilicatebased crown.

(3) Duolink always presented higher DC regardless of the ceramic composition and curing protocol.
